# Supplementary material for: Shared decision-making and deprescribing to support anti-thrombotic therapy (dis)continuance for persons living with cancer in their last phase of life: A realist synthesis
Source: PLoS Med. 2025 Aug 25;22(8):e1004663. doi: 10.1371/journal.pmed.1004663 (PMC12410886; doi:10.1371/journal.pmed.1004663)
Supplement: S1 File — (DOCX) [file pmed.1004663.s001.docx]

**SERENITY WP1 Initial Programme Theories**

**IPT 1: (Clinical Equipoise)** If clinicians have clinical equipoise and are comfortable managing the uncertainty of an end-of-life prognosis, and if they come to a balanced understanding of the harms of continued prescribing versus harms of stopping, they will feel more willing to engage in genuine forms of shared decision making with patients. They will be honest with patients about the uncertainty and invite patients to have their say on the course of action to be taken with regard to treatment options. Such actions will increase the likelihood patients will suffer less and have improved quality of life at the end of life.

**IPT 2:** **(Clinician Recognition of End-of-Life Transition)** If clinicians become more competent in recognizing when a person is approaching the end of life, they will be more ready to engage in shared decision making with the patient and convey needed information about treatment benefits and drawbacks to improve their quality of life through to the end of life.

**IPT 3:** **(Clinician Awareness of Patient Readiness)** If clinicians are aware of the level of readiness of patients to engage in SDM around their end-of-life care and dispel preconceived notions that patients across the board do not want to engage in such conversations, they will be more motivated to initiate SDM conversations with patients and their families.

**IPT 4:** **(Changing Impact of Medications)** As physiological changes occur in the body through the process of dying, medications that have been prescribed for a long time (such as for osteoporosis or heart disease) and which did not cause side-effects previously, may increase risks of side-effects for the current physiology (e.g., bleeding events). Furthermore, clinicians may not realize that it is the medications creating such symptoms and erroneously attribute such events to the dying process.

**IPT 5:** **(The Meaning of Medications for Patients)** Medications meant to delay cancer progressions may be difficult to de-prescribe because they offer hope to patients that they may be cured of their cancer, or have their extended months or years to live as a result. Clinicians may avoid conversations around deprescribing such medications and default to an optimistic view because of the uncertainty on prognosis, even if the reality is that the patient is not going to survive, and their life will not be prolonged in a significant way due to the use of such medications.

**IPT 6: (Broadening the Conversation with Patients)** If clinicians situate their conversations about deprescribing in a wider discussion with patients and families regarding their values, beliefs and desires for care, (as opposed to very focused discussion about medication review), then patients will better understand the benefit of deprescribing and will not feel the doctor is giving up on them or trying to speed up their death. Clinicians will also reduce their fear that they are upsetting and burdening patients with complex medical information

**IPT 7:** **(Clinician Comfort in Early Initiation of Palliative Conversation)** If clinicians overcome their fear of conversing with relatively healthy patients with a terminal diagnosis regarding how long they are expected to live, about how long a patient is expected to live while they are still relatively healthy, they will engage in SDM and realize that most patients feel relieved at having such conversations. Such actions will lead to improved SDM and optimal prescribing as patients move toward the last phase of life

**IPT 8: (Clinician Awareness of Changes to Patient Readiness for SDM)** If clinicians realize that the initial limited engagement of patients and families in term of their involvement in medical decisions is temporary (due to the need for processing a difficult diagnosis) and that volition typically increases over time, they will become more sensitive to knowing the right moment to engage in SDM conversation about values (not too early, not too late). This will allow patients and families to engage in the process when they are psychologically ready to and will increase chances of optimal deprescribing and improved quality of life in the last phase of life.

**IPT 9: (Clinician Empathic Communication)** If clinicians are trained in effective, empathic communication with patients around end-of-life care needs then they will overcome their fear and avoidance of such communication and engage patients in SDM before a life-threatening crisis occurs. Such early efforts will increase the chances that patients will be able to digest the information they receive, and have care be aligned with their goals and values.

**IPT 10:** **(Specialized Language for Communicating Deprescribing)** If clinicians use specialized language in conversations with patients regarding treatment options to maximize benefit and limit harms (rather than simply ‘stopping medications’) then patients will interpret these deprescribing efforts as life enhancing/improving rather than feeling abandoned by the clinician. Some patients may also feel relieved by conversations around deprescribing because they want to stop taking the medications but did not realize they could raise their concerns with the clinician. Using specialized language may also increase the confidence of clinicians in engaging in genuine forms of SDM with patients.

**IPT 11: (Hope for Survival is Lost when Deprescribing Happens without SDM)** If clinicians remove medications without a genuine SDM conversation with the patient in the last phase of life (and their family) then patients may feel given up on or worse may feel the clinician is removing medications to hasten the end-of-life process

**IPT 12: (Shared Decision Making leads to Optimal Deprescribing)** If deprescribing is done with genuine SDM, then both clinician and patient/family will feel at ease with the decision. Patients will understand that medication removal will improve quality of life, and clinicians will feel confident in their approach knowing they are doing what the patient prefers

**IPT 13: (Clinician Concern for Offending Original Prescriber)** If a potentially inappropriate medication (PIM) is identified during a medication review with the patient, then the clinician may have reservations about deprescribing if they were not the original prescriber because of a fear they lack the competencies to determine best course of action. This issue is exacerbated if the original prescriber is a specialist (for example oncologist) and the medication reviewer is not specifically trained in that area of clinical practice (e.g., geriatric specialist). Considerations for de-prescribing may also result in differences of opinion across the healthcare team, leading again to prescribing inertia and maintaining the *status quo* on medication prescriptions.

**IPT 14: (Clinician Assumptions about Patient Health Literacy)** If clinicians do not engage patients in shared decision making, they may assume that the patient lack of engagement and deference means a lack of health literacy and ability to engage. This may result in poor outcomes for patients such as default to pro-active treatment, as their values and goals for care have not been explored in relation to treatment and medication review

**IPT 15: (PIMS List Inspire Confidence for Deprescribing)** If clinicians are trained to use PIMs lists for de-prescribing potentially inappropriate medications, they will realize which medications may be most risky to continue for palliative cancer patients and increase their confidence in deprescribing as they can refer to the guidance offered in the PIMs list to justify their decision.
