# Supplementary material for: Shared decision-making and deprescribing to support anti-thrombotic therapy (dis)continuance for persons living with cancer in their last phase of life: A realist synthesis
Source: PLoS Med. 2025 Aug 25;22(8):e1004663. doi: 10.1371/journal.pmed.1004663 (PMC12410886; doi:10.1371/journal.pmed.1004663)
Supplement: S2 File — (DOCX) [file pmed.1004663.s002.docx]

**Search narrative**

**SCOPING searches (May/June 2022)**The main search concepts to address the review questions (e.g. palliative care, deprescribing, shared decision-making/goal concordant care, anti-thrombotic medication) were researched by examining the indexing of known relevant studies and using tools such as PubMed PubReminer to analyse focused search results.

Existing validated search filters for palliative care (Sladek 2007, Rietjens 2019) were identified and the methods of existing systematic reviews on deprescribing in palliative cancer patients (Lindsay 2014, Brokaar 2022 and Merendonk 2022) were reviewed to help develop an initial search for deprescribing which was tested against a set of 47 known relevant results from systematic reviews and edited as necessary to improve recall.

We looked for SDM theory: general search on Google and reviewed the first 4 pages of results, searched Medline and Epistemonikos for systematic reviews and then did a very focused search on Web of Science and Scopus (words in title only).

**INITIAL searches (October 2022)**An initial 3 concept search combining (SDM or goal concordant care) AND palliative care AND deprescribing retrieved around 350 results in Medline. A decision was made to broaden the search to **palliative care AND (SDM/goal concordant care or deprescribing**) to avoid missing studies on SDM/GCC not focused on deprescribing and to limit to post 2010 results. Search sent to JJ and MP before running.

This search was an exploratory, Medline only search to quickly review results to identify and fix any potential issues with the search BEFORE running the multi-database search for Stage 1 (See document Medline exploratory search 1). We removed child and animal only studies from search (protocol is 18+ only population). Search strategies for all databases can be found below

**MAIN DATABASE SEARCH 17-18 October 2022 (Stage 1 search, Step A)**Feedback from JJ confirmed to go ahead with full database search. Main database searches translated and run in:

| **Search date** | **Database** | **Platform** | **Search yield** |
| --- | --- | --- | --- |
| 17^th^ October 2022 | Medline All | OVID | 8029 |
| 17^th^ October 2022 | Embase | OVID | 6577 |
| 17^th^ October 2022 | APA PsycInfo | OVID | 2209 |
| 17^th^ October 2022 | CINAHL | Ebsco | 6895 |
| 17^th^ October 2022 | Cochrane Database of Systematic Reviews | The Cochrane Library | 110 |
| 17^th^ October 2022 | Cochrane Central Register of Controlled Trials | The Cochrane Library | 1475 |
| 17^th^ October 2022 | ASSIA | Proquest | 462 |
| 17^th^ October 2022 | SCI-EXPANDED, SSCI, AHCI, CPCI-S, CPCI-SSH, ESCI | Web of Science | 278 |
| 18^th^ October 2022 | Epistemonikos* | [https://www.epistemonikos.org/#](https://www.epistemonikos.org/) | 9 |
| 18^th^ October 2022 | Google Scholar check* |  | 66 |
|  |  |  |  |

*Very focused supplementary title only searches to identify unique studies not already found (Google Scholar results were mainly dissertations)

Record of results from each database saved in a spreadsheet (Serenity search results.xls) but in brief, 26110 results before duplicates removed and 17036 after duplicates removed. A RIS file of these results was exported from Endnote and imported into Covidence (SERENITY Realist Review Search 1). After a trial of screening, to fully utilise the reverse chronological screening method, instead Rayyan was used as a screening tool to fill “Quotas” of topics.

**Stage 1 search, Step B (1^st^ June 2023)**Following review team discussions, the results of Search 1, Step A were searched within the Endnote database for the following three themes:

- Organisational factors in deprescribing (MeSH term for organizational culture, organizational factors)
- Moral distress in difficult situations (including avoidance, truth disclosure, moral distress difficult conversations/situations)
- Anti-thrombotic therapy and deprescribing

257 results from this combined supplementary search were loaded into a Rayyan project for further reverse chronology screening

**Main database search strategies for all databases:**

SERENITY: Search strategies for all databases

**OVID Medline**

1. Decision Making, Shared/

2. (shared adj2 decision making).ti,ab,kw.

3. 1 or 2 [shared decision making]

4. exp *Decision Making/

5. (prefer* or decision* or decide* or deciding or choice or choose).ti,kw.

6. *decision support techniques/

7. (decision adj3 making).ti,ab,kw,kf.

8. Choice Behavior/

9. or/4-8 [ decision making]

10. Physician-Patient Relations/

11. Patient Preference/

12. Patient Participation/

13. Patient-Centered Care/

14. ((patient* or person) adj2 (centered or centred)).ti,ab,kw.

15. or/10-14 [patient focus]

16. 9 and 15 [decision making AND patient focus]

17. 3 or 16 [combined SDM concept]

18. ((goal* or prefer* or value* or wish* or request* or record* or statement* or report*) adj2 (align* or respect* or match* or "consistent" or concord* or complian*)).ti,ab,kw.

19. exp Patient Care Planning/

20. 15 or 19

21. 18 and 20

22. (goal-concordant or goals of care or care goals).ti,ab,kw. or goals/

23. 21 or 22 [goal concordant care concept]

24. 17 or 23 [SDM or goal concordant care concept]

25. deprescriptions/

26. Inappropriate Prescribing/

27. Potentially Inappropriate Medication List/

28. (deprescrib* or de-prescrib* or deprescription* or de-prescription* or STOPPFrail or OncPal).ti,ab,kw,kf.

29. ((inappropriate or unnecessary or unneeded or over or futile) adj3 (prescript* or prescrib* or medicat* or drug*)).ti,ab,kw,kf.

30. ((medication* or drug* or therapy* or treatment*) adj4 (reduction or discontinu* or withdraw* or stop*)).ti,ab,kw,kf.

31. Medical Futility/

32. or/25-31 [deprescribing concept]

33. exp advance care planning/

34. exp attitude to death/

35. exp bereavement/

36. death/

37. hospices/ or "Hospice and Palliative Care Nursing"/

38. life support care/

39. palliative care/ or Palliative Medicine/

40. exp terminal care/ or respite care/

41. terminally ill/

42. palliat$.af.

43. hospice$.af.

44. (terminal care or respite care).af.

45. or/33-44

46. journal of palliative care.jn.

47. journal of palliative medicine.jn.

48. hospice journal physical psychosocial & pastoral care of the dying.jn.

49. supportive care in cancer.jn.

50. palliative medicine.jn.

51. palliative & supportive care.jn.

52. journal of supportive oncology.jn.

53. journal of social work in end of life & palliative care.jn.

54. journal of pain & symptom management.jn.

55. journal of pain & palliative care pharmacotherapy.jn.

56. international journal of palliative nursing.jn.

57. death studies.jn.

58. death education.jn.

59. american journal of hospice care.jn.

60. american journal of hospice & palliative medicine.jn.

61. omega journal of death & dying.jn.

62. or/46-61

63. 45 or 62

64. bereave*.mp.

65. attitude to death.mp.

66. end of life.af.

67. Advance* Care.af.

68. ((advanced or terminal*) adj (ill* or disease)).ti,ab,kw.

69. supportive care.ti,ab,kw.

70. dying.ti,ab,kw.

71. "last year of life".ti,ab,kw.

72. (limited life adj (expectanc* or span*)).ti,ab,kw. or life-limiting.mp.

73. or/64-72

74. 63 or 73 [palliative care concept]

75. 24 and 32 and 74 [SDM or goal concordant care AND deprescribing AND palliative care]

76. 74 and 24 [palliative care AND SDM/goal concordant care]

77. 74 and 32 [palliative care AND deprescribing]

78. 76 or 77 [palliative care AND SDM or deprescribing]

79. (animals not humans).sh.

80. 78 not 79 [removes animal only studies]

81. limit 80 to yr="2010 -Current"

82. (exp infant/ or exp child/ or adolescent/) not exp adult/

83. 81 not 82 [removes MeSH indexed child only studies]

**EMBASE via OVID**

1. shared decision making/

2. (shared adj2 decision making).ti,ab,kw.

3. 1 or 2 [shared decision making]

4. exp *decision making/

5. (prefer* or decision* or decide* or deciding or choice or choose).ti,kw.

6. (decision adj3 making).ti,ab,kw,kf.

7. 4 or 5 or 6 [ decision making]

8. exp *professional-patient relationship/

9. *patient preference/

10. patient participation/

11. ((patient* or person) adj2 (centered or centred)).ti,kw.

12. 8 or 9 or 10 or 11 [patient focus]

13. 7 and 12 [decision plus patient focus]

14. 3 or 13 [SDM concept]

15. ((goal* or prefer* or value* or wish* or request* or record* or statement* or report*) adj2 (align* or respect* or match* or "consistent" or concord* or complian*)).ti,ab,kw.

16. patient care planning/

17. 12 or 16

18. 15 and 17

19. (goal-concordant or goals of care or care goals).ti,ab,kw.

20. 18 or 19 [goal concordant care]

21. 14 or 20 [SDM or goal concordant care concept]

22. deprescription/

23. unnecessary prescribing/ or inappropriate polypharmacy/ or overprescribing/ or prescribing cascade/

24. potentially inappropriate medication/

25. prescribing error/ or unnecessary prescribing/

26. (deprescrib* or de-prescrib* or deprescription* or de-prescription* or STOPPFrail or OncPal).ti,ab,kw,kf.

27. ((inappropriate or unnecessary or unneeded or over or futile) adj3 (prescript* or prescrib* or medicat* or drug*)).ti,ab,kw,kf.

28. ((medication* or drug* or therapy* or treatment*) adj2 (reduction or discontinu* or withdraw* or stop*)).ti,ab,kw,kf.

29. or/22-28 [deprescribing concept]

30. advance care planning/

31. attitude to death/

32. bereavement/

33. death/

34. hospice/

35. exp palliative therapy/

36. respite care/

37. terminal care/ or hospice care/

38. exp terminally ill patient/

39. palliat$.af.

40. hospice$.af.

41. (terminal care or respite care).af.

42. supportive care.ti,ab,kw.

43. bereave$.mp.

44. attitude to death.mp.

45. end of life.af.

46. ((advanced or terminal* or critical*) adj (ill* or disease)).ti,ab,kw.

47. Advance* Care.af.

48. (limited life adj (expectanc* or span*)).ti,ab,kw. or life-limiting.mp.

49. "last year of life".ti,ab,kw.

50. dying.ti,ab,kw.

51. or/30-50 [palliative concept]

52. 51 and 21 [palliative care AND SDM/goal concordant care]

53. 51 and 29 [palliative care and deprescribing]

54. 52 or 53 [palliative care AND (SDM or deprescribing)]

55. (animal$ not human$).sh,hw.

56. exp child/ not exp adult/

57. 54 not (55 or 56) [remove animal and child only studies]

58. limit 57 to conference abstract status

59. 57 not 58 [remove conf abstracts]

60. limit 59 to yr="2010 -Current"

**APA PsycInfo via OVID**

1. (shared adj2 decision making).ti,ab,kp.

2. decision making/

3. (prefer* or decision* or decide* or deciding or choice or choose).ti,kp.

4. 2 or 3

5. exp Client Participation/

6. exp Patient Centered Care/

7. ((patient* or person) adj2 (centered or centred)).ti,ab,kp.

8. 5 or 6 or 7

9. 4 and 8

10. 1 or 9 [SDM]

11. goals/

12. ((goal* or prefer* or value* or wish* or request* or record* or statement* or report*) adj2 (align* or respect* or match* or "consistent" or concord* or complian*)).ti,ab,kp.

13. 11 or 12

14. 8 and 13 [patient focused AND goals]

15. (goal-concordant or goals of care or care goals).ti,ab,kp.

16. 14 or 15 [GCC]

17. 10 or 16 [SDM or goal concordant care]

18. (deprescrib* or de-prescrib* or deprescription* or de-prescription* or STOPPFrail or OncPal).ti,ab,kp.

19. ((inappropriate or unnecessary or unneeded or over or futile) adj3 (prescript* or prescrib* or medicat* or drug*)).ti,ab,kp.

20. ((medication* or drug* or therapy* or treatment*) adj4 (reduction or discontinu* or withdraw* or stop*)).ti,ab,kp.

21. exp Treatment Withholding/

22. or/18-21 [deprescribing]

23. exp Advance Directives/ or exp Terminally Ill Patients/ or exp Palliative Care/ or exp "Death and Dying"/

24. hospice/

25. (palliat* or hospice* or terminal care).af.

26. bereave*.mp.

27. attitude to death.mp.

28. end of life.af.

29. Advance* Care.af.

30. ((advanced or terminal*) adj (ill* or disease)).ti,ab.

31. supportive care.ti,ab.

32. dying.ti,ab.

33. last year of life.ti,ab.

34. (limited life adj (expectanc* or span*)).ti,ab. or life-limiting.mp.

35. or/23-34 [palliative care]

36. 35 and 17 [Palliative care AND SDM/goal concordant care]

37. 35 and 22 [Palliative care and deprescribing]

38. 36 or 37

39. limit 38 to yr="2010 -Current"

40. (animal not human).po.

41. 39 not 40

**CINAHL Complete via EBSCO**

| **#** | **Query** |
| --- | --- |
| S1 | (MH "Decision Making, Shared") or (MH "Decision Making, Patient+") |
| S2 | (MH "Decision Making+") |
| S3 | (MH "Professional-Patient Relations+") |
| S4 | (MH "Patient Preference") |
| S5 | TI ( prefer* or decision* or decide* or deciding or choice or choose ) OR MW ( prefer* or decision* or decide* or deciding or choice or choose ) |
| S6 | TI decision N2 making OR AB decision N2 making |
| S7 | (MH "Consumer Participation") |
| S8 | (MH "Patient Centered Care") |
| S9 | TI ( ((patient* or person) N2 (centered or centred)) ) OR AB ( ((patient* or person) N2 (centered or centred)) ) |
| S10 | TI shared decision making OR AB shared decision making |
| S11 | S2 OR S6 |
| S12 | S3 OR S4 OR S7 OR S8 OR S9 |
| S13 | S11 AND S12 |
| S14 | S1 OR S10 OR S13 |
| S15 | TI ( goal-concordant or "goals of care" or "care goals" ) OR AB ( goal-concordant or "goals of care" or "care goals" ) |
| S16 | (MH "Goals and Objectives") and S12 |
| S17 | S14 OR S15 OR S16 |
| S18 | (MH "Deprescribing") |
| S19 | (MH "Inappropriate Prescribing") |
| S20 | TI ( deprescrib* or de-prescrib* or deprescription* or de-prescription* or STOPPFrail or OncPal ) OR AB ( deprescrib* or de-prescrib* or deprescription* or de-prescription* or STOPPFrail or OncPal ) |
| S21 | TI ( ((inappropriate or unnecessary or unneeded or over or futile) N3 (prescript* or prescrib* or medicat* or drug*)) ) OR AB ( ((inappropriate or unnecessary or unneeded or over or futile) N3 (prescript* or prescrib* or medicat* or drug*)) ) |
| S22 | S18 OR S19 OR S20 OR S21 |
| S23 | ( (MH "Terminal Care+") or (MH "Palliative Care") or (MH "Attitude to Death") or (MH "Advance Care Planning") or (MH "Respite Care") or (MH "Hospices") or (MH "Life Support Care") ) OR TI ( bereave* or hospice* or "end of life" of "terminally ill" or palliat* ) OR AB ( bereave* or hospice* or "end of life" of "terminally ill" or palliat* ) |
| S24 | TI life-limiting OR AB life-limiting |
| S25 | TI ( ((advanced or terminal* or critical*) n1 (ill* or disease)) ) OR AB ( ((advanced or terminal* or critical*) n1 (ill* or disease)) ) |
| S26 | TI ( (limited life N1 (expectanc* or span*)) ) OR AB ( (limited life N1 (expectanc* or span*)) ) |
| S27 | S23 OR S24 OR S25 OR S26 |
| S28 | S17 OR S22 |
| S29 | S27 AND S28 |
| S30 |  |
| S31 | S29 AND S30 |

**CDSR and CENTRAL via The Cochrane Library**

ID Search

#1 MeSH descriptor: [Decision Making, Shared] 2 tree(s) exploded

#2 MeSH descriptor: [Decision Making] explode all trees

#3 MeSH descriptor: [Physician-Patient Relations] explode all trees

#4 MeSH descriptor: [Patient Preference] explode all trees

#5 MeSH descriptor: [Choice Behavior] explode all trees

#6 MeSH descriptor: [Patient Participation] explode all trees

#7 MeSH descriptor: [Patient-Centered Care] explode all trees

#8 (prefer* or decision* or decide* or deciding or choice or choose):ti

#9 (prefer* or decision* or decide* or deciding or choice or choose):kw

#10 decision near/3 making

#11 (((patient* or person) near/2 (centered or centred))):ti,ab,kw

#12 (shared decision making):ti,ab,kw

#13 #1 or #12

#14 #2 or #5 or #8 or #9 or #10

#15 #3 or #4 or #6 or #7 or #11

#16 #14 and #15

#17 #13 or #16

#18 (goal-concordant or "goals of care" or "care goals"):ti,ab,kw

#19 (((goal* or prefer* or value* or wish* or request* or record* or statement* or report*) NEAR/2 (align* or respect* or match* or "consistent" or concord* or complian*))):ti,ab,kw

#20 MeSH descriptor: [Patient Care Planning] explode all trees

#21 #15 or #20

#22 #19 and #21

#23 #18 or #22

#24 #17 or #23

#25 MeSH descriptor: [Deprescriptions] explode all trees

#26 MeSH descriptor: [Inappropriate Prescribing] explode all trees

#27 MeSH descriptor: [Potentially Inappropriate Medication List] explode all trees

#28 (deprescrib* or de-prescrib* or deprescription* or de-prescription* or STOPPFrail or OncPal):ti,ab,kw

#29 (((inappropriate or unnecessary or unneeded or over or futile) NEAR/3 (prescript* or prescrib* or medicat* or drug*))):ti,ab,kw

#30 MeSH descriptor: [Medical Futility] explode all trees

#31 ((medication* or drug* or therapy* or treatment*) NEAR/2 (reduction or discontinu* or withdraw* or stop*))

#32 [89-#31]

#33 #24 or #32

#34 MeSH descriptor: [Advance Care Planning] explode all trees

#35 MeSH descriptor: [Attitude to Death] explode all trees

#36 MeSH descriptor: [Bereavement] explode all trees

#37 MeSH descriptor: [Death] this term only

#38 MeSH descriptor: [Hospices] this term only

#39 MeSH descriptor: [Life Support Care] this term only

#40 MeSH descriptor: [Palliative Care] explode all trees

#41 MeSH descriptor: [Respite Care] explode all trees

#42 MeSH descriptor: [Terminal Care] explode all trees

#43 MeSH descriptor: [Terminally Ill] explode all trees

#44 (palliat* or hospice*):ti,ab,kw

#45 ((terminal or supportive) next care):ti,ab,kw

#46 (respite next care):ti,ab,kw

#47 (bereave* or dying):ti,ab,kw

#48 ("attitude to death"):ti,ab,kw

#49 (((advanced or terminal* or critical*) next (ill* or disease))):ti,ab,kw

#50 (advance* next care):ti,ab,kw

#51 ("last year of life" or (limited next (expectanc* or span*)) or life-limiting):ti,ab,kw

#52 #34 or #35 or #36 or #37 or #38 or #39 or #40 or #41 or #42 or #43 or #44 or #45 or #46 or #47 or #48 or #49 or #50 or #51

#53 #33 and #52

**EPISTEMONIKOS database via** [**https://www.epistemonikos.org/#**](https://www.epistemonikos.org/)

title:(palliative OR hospice OR end of life) AND title:(deprescribing OR deprescription OR goals of care OR goal concordant OR shared decision making)

**Web of Science Core Collection (Including SCI-EXPANDED, SSCI, AHCI, CPCI)**

**(palliat* or hospice or end of life or life limiting)** (Title) and **shared decision making or goal concordance or goal concordant care or goals of care or deprescribing or deprescription or inappropriate prescribing or inappropriate prescriptions** (Title)

**ASSIA via PROQUEST**

(noft("shared decision making" OR "shared decision-making" OR "goals of care" OR "goal-concordant care" OR "goal concordant care" OR "goal concordance") OR (noft(deprescrib* OR de-prescrib* OR deprescription* OR de-prescription* OR STOPPFrail OR OncPal) OR noft((inappropriate OR unnecessary OR futile) NEAR/3 (prescription* OR medication* OR drug* OR prescribing)))) AND (MAINSUBJECT.EXACT("Palliative care") OR MAINSUBJECT.EXACT("Hospices") OR MAINSUBJECT.EXACT("End of life decisions") OR MAINSUBJECT.EXACT.EXPLODE("Terminally ill people") OR noft(palliat* OR hospice* OR terminally ill) OR noft("end of life" OR "last year of life") OR noft(life-limiting OR "limited life expectancy")) AND yr(2010-2022)

**Google Scholar Check:**

allintitle: end of life deprescribing

allintitle: end of life shared decision making

| **Section/topic** | **#** | **Checklist item** | | | **Location(s) Reported** |
| --- | --- | --- | --- | --- | --- |
| **INFORMATION SOURCES AND METHODS** | | | | | |
| Database name | 1 | Name each individual database searched, stating the platform for each. | | | Page 7 and Appendix 1 |
| Multi-database searching | 2 | If databases were searched simultaneously on a single platform, state the name of the platform, listing all of the databases searched. | | | N/A |
| Study registries | 3 | List any study registries searched. | | | N/A |
| Online resources and browsing | 4 | Describe any online or print source purposefully searched or browsed (e.g., tables of contents, print conference proceedings, web sites), and how this was done. | | | Page 7 and Appendix 1 |
| Citation searching | 5 | Indicate whether cited references or citing references were examined, and describe any methods used for locating cited/citing references (e.g., browsing reference lists, using a citation index, setting up email alerts for references citing included studies). | | | N/A |
| Contacts | 6 | Indicate whether additional studies or data were sought by contacting authors, experts, manufacturers, or others. | | | Page 10 (expert solicitation) |
| Other methods | 7 | Describe any additional information sources or search methods used. | | | Page 7 (Stage 1 Step B) |
| **SEARCH STRATEGIES** | | | | | |
| Full search strategies | 8 | Include the search strategies for each database and information source, copied and pasted exactly as run. | | | Appendix 1 |
| Limits and restrictions | 9 | Specify that no limits were used, or describe any limits or restrictions applied to a search (e.g., date or time period, language, study design) and provide justification for their use. | | | Page 7 |
| Search filters | 10 | Indicate whether published search filters were used (as originally designed or modified), and if so, cite the filter(s) used. | | | Page 7 |
| Prior work | 11 | Indicate when search strategies from other literature reviews were adapted or reused for a substantive part or all of the search, citing the previous review(s). | | | Page 7 |
| Updates | 12 | Report the methods used to update the search(es) (e.g., rerunning searches, email alerts). | | | N/A |
| Dates of searches | 13 | For each search strategy, provide the date when the last search occurred. | | | Page 7 |
| **PEER REVIEW** | | | | | |
| Peer review | 14 | Describe any search peer review process. | | | Page 7 |
| **MANAGING RECORDS** | | | | | |
| Total Records | 15 | Document the total number of records identified from each database and other information sources. | | | Appendix 1 |
| Deduplication | 16 | Describe the processes and any software used to deduplicate records from multiple database searches and other information sources. | | | Appendix 1 |
|  |  |  | | |  |
| PRISMA-S Checklist (Rethlefsen ML, Kirtley S, Waffenschmidt S, Ayala AP, Moher D, Page MJ, Koffel JB; PRISMA-S Group. PRISMA-S: an extension to the PRISMA Statement for Reporting Literature Searches in Systematic Reviews. Syst Rev. 2021;10(1):39. doi: [10.1186/s13643-020-01542-z](https://doi.org/10.1186/s13643-020-01542-z)) | | | | |  |
|  | | | | |  |
|  | | |  |  |  |
