# Supplementary material for: Shared decision-making and deprescribing to support anti-thrombotic therapy (dis)continuance for persons living with cancer in their last phase of life: A realist synthesis
Source: PLoS Med. 2025 Aug 25;22(8):e1004663. doi: 10.1371/journal.pmed.1004663 (PMC12410886; doi:10.1371/journal.pmed.1004663)
Supplement: S3 File — (DOCX) [file pmed.1004663.s003.docx]

| **Patient and Public Involvement** | | | |
| --- | --- | --- | --- |
|  | Meeting held in the: | # of attendees | Questions and Theory Statements Explored: |
| Meeting #1 | 5^th^ month | 3 | - Why don’t doctors deprescribe? - Why don’t patients and their families advocate for deprescribing? - What is important to make sure patients/families are involved in decisions about deprescribing? - Example of efforts to improve deprescribing |
| Meeting #2 | 10^th^ month | 6 | - Widening the conversation with patients about preferences and values - Multi-disciplinary team working - Empowering patients in advance - Differences in cultural views on death |
| SERENITY Consortium | | | |
| Meeting #1 | 5^th^ month | 23 | - What is important for understanding the clinician’s role in deprescribing ATT at end of life? - How can patients and families be empowered to improve optimization of deprescribing efforts? - What recommendations do you have to focus the realist synthesis on ATT deprescribing and shared decision-making? |
| Meeting #2 | 10^th^ month | 17 | - What is important to understand about how doctors widen the conversation to include personal values, perspectives and belief systems of patients? - What is important to understand about improving multidisciplinary team working for optimal deprescribing? - What is important to understand about preparing patients in advance of meetings with healthcare providers? - How can clinicians be responsive to cultural differences when it comes to deprescribing medications through shared decision-making with patients? - How can healthcare organizations and practitioners navigate legal contexts and improve organizational policies that incentivize a shared decision-making approach to optimize deprescribing efforts? |
