# Supplementary material for: Shared decision-making and deprescribing to support anti-thrombotic therapy (dis)continuance for persons living with cancer in their last phase of life: A realist synthesis
Source: PLoS Med. 2025 Aug 25;22(8):e1004663. doi: 10.1371/journal.pmed.1004663 (PMC12410886; doi:10.1371/journal.pmed.1004663)
Supplement: S4 File — (DOCX) [file pmed.1004663.s004.docx]

| **Authors** | **Title** | **Year** | **Country** | **Study Design:** | **Discipline** | **Focus on Deprescribing (DP), Shared Decision-Making (SDM) or both?** | **Stakeholder being investigated: e.g., clinicians, patients, other** |  |
| --- | --- | --- | --- | --- | --- | --- | --- | --- |
| [35] Shrestha S., Poudel, A., Reeve, E. Et al | Development and validation of a tool to understand healthcare professionals’ attitudes towards deprescribing (HATD) in older adults with limited life expectancy | 2022 | Australia | Survey | Palliative | DP | Clinicians and wider healthcare staff (doctors, nurses and pharmacists) |  |
| [36] Lundby C., Graabaek T., Ryg J. et al | Health care professionals' attitudes towards deprescribing in older patients with limited life expectancy: A systematic review | 2019 | Denmark | Systematic review and meta-analysis | Palliative | Both | Care providers, wider healthcare staff and care organisations |  |
| [37] Geijteman, E. C, Kuip, E. J., Oskam, J. Et al | Illness trajectories of incurable solid cancers | 2024 | Netherlands | Commentary | Palliative Oncology | DP | Clinicians |  |
| [38] Hedman C., Frisk G., Bjorkhem-Bergman L. (2022) | Deprescribing in Palliative Cancer Care | 2022 | Sweden | Systematic Review | Palliative Medicine | DP | Clinicians |  |
| [39] Dodek P., Jameson K., Chevalier J. et al | New approach to assessing and addressing moral distress in intensive care unit personnel: a case study | 2022 | Canada | Case report | Intensive Care | SDM | Clinicians and wider healthcare staff |  |
| [40] McGraw, C. | Involving older people in decisions about deprescribing in end-of-life care | 2022 | United Kingdom | Commentary | Palliative Nursing | SDM | Nurses |  |
| [41] Brown J., Myers H., Eng D. et al | Evaluation of the ‘Talking Together’ simulation communication training for ‘goals of patient care’ conversations: a mixed—methods study in five metropolitan public hospitals in Western Australia | 2022 | Australia | Mixed-methods study | Oncology | SDM | Patients, family/carers, clinicians | |
| [42] Curtin D., Gallagher P., O’Mahony D. | Deprescribing in older people approaching end-of-life: development and validation of STOPPFrail version 2 | 2021 | Ireland | Systematic Review and Delphi Consensus Methodology | Geriatric Medicine | DP | Clinicians |  |
| [43] Jimenez G., Tan W., Virk A. et al | Overview of Systematic Reviews of Advanced Care Planning: Summary of Evidence and Global Lessons | 2018 | Singapore | Systematic Review | Palliative Medicine | SDM | Clinicians and Patients | |
| [44] Fortin G., Dumont S. | Goals of Care Conversations at the End of Life: Perceived Impact of an Interprofessional Training Session on Professional Practices | 2021 | Canada | Interviews | Palliative | Both | Care providers, patients, healthcare establishments |  |
| [45] Singh-Carlson S., Reynolds G., Wu S. | The Impact of Organizational Factors on Nurses' Knowledge, Perceptions, and Behaviors Around Advance Care Planning | 2020 | USA | Survey analysis | Nursing | SDM | Nurses, oncology organisations and patients |  |
| [46] Bleicher J., Grudziak J., Lambert LA. et al | Drivers of Moral Distress in Surgical Intensive Care Providers: A Mixed Methods Study | 2021 | USA | Mixed methods study | Care of SICU patients | Shared decision making | Clinicians and nurses |  |
| [47] Statham, E.E., & Marron J.M. | Counterpoint: Provider moral distress in end‐of‐life oncology care is a moral crutch | 2017 | USA | Narrative review | Oncology patients at EoL | Shared decision making | Care providers and patients |  |
| [48] Prentice T., Gillam L. | Can the Ethical Best Practice of Shared Decision-Making lead to Moral Distress? | 2018 | Australia | Commentary | General | SDM | Clinicians and nurses |  |
| [49] Bader C. S., Herschkopf MD. | Trainee Moral Distress in Capacity Consultations for End-of-Life Care | 2019 | USA | Case report | Palliative | SDM | Clinicians |  |
| [50] Choi HR., Kang SW., Rodgers S. et al | Nurse's, physician's and family member's experiences of withholding or withdrawing life-sustaining treatment process in an intensive care unit | 2022 | South Korea | Interview study | Palliative | Both | Clinicians, wider healthcare staff (nurses) and patients |  |
| [51] Kim K., Heinze K., Xu J. et al | Theories of health care decision making at the end of life: A meta-ethnography | 2018 | United States | Meta-ethnography | Nursing | Both | Clinicians and Patients |  |
| [52] Johnson, R. F. | “Farewell” to Prognosis in Shared Decision-Making | 2020 | USA | Commentary | Palliative | SDM | Clinicians and Patients |  |
| [53] Steiner J., Patton K., Prutkin, J et al | Moral Distress at the End of a Life: When Family and Clinicians Do Not Agree on Implantable Cardioverter-Defibrillator Deactivation | 2018 | USA | Case report | Palliative | Both | Clinicians, patients and families |  |
| [54] Brett AS., McCullough LB. | Getting Past Words: Futility and the Professional Ethics of Life-Sustaining Treatment | 2017 | USA | Narrative review | Palliative | Both | Care providers, patients |  |
| [55] Aghabarary M., Nayeri ND. | Reasons behind providing futile medical treatments in Iran: A qualitative study | 2017 | Iran | Qualitative exploratory study | Palliative | Both | Care providers, patients, family members, organisations |  |
| [56] Relias Media Group | Physicians reported moral distress about surrogate decision-makers | 2020 | USA | Commentary | Palliative | SDM | Clinicians |  |
| [57] Epstein A., Riley M., Nelson J. et al | Goals of care documentation by medical oncologists and oncology patient end-of-life care outcomes | 2022 | USA | Observational retrospective cohort study | Palliative Oncology | SDM | Patients, care providers |  |
| [58] Button E., Cardona M., Huntley K. et al | Clinicians’ Understanding of Preferences and Values of People with Hematological Malignancies at the End of Life: Concurrent Surveys | 2022 | Australia | Surveys | Palliative | SDM | Clinicians, patients |  |
| [59] Wasp G., Knutzen K., Murray G. et al | Systemic Therapy Decision Making in Advanced Cancer: A Qualitative Analysis of Patient-Oncologist Encounters | 2021 | Lebanon | Qualitative analysis | Palliative Oncology | Both | Clinicians, patients |  |
| [60] Yeh A., Sun A., Chernicoff H. et al | Polypharmacy in Hospice and Palliative Care | 2022 | USA | Summary review | Palliative | Both | Clinicians and patients |  |
| [61] Braun U., Ford M., Beyth J. et al | The physician’s professional role in end of life decision-making: Voices of racially and ethnically diverse physicians | 2010 | USA | Qualitative Study | Palliative | Both | Clinicians and organisations |  |
| [62] Reeve J., Maden M., Hill R. et al | Deprescribing medicine in older living with multimorbidity and polypharmacy: the TAILOR evidence synthesis | 2022 | United Kingdom | Scoping and Realist Review | Palliative Medicine | DP | Clinicians and Patients |  |
| [63] Casey M., Price L., Markwalter D. et al | Advance Care Planning for Emergency Department Patients With COVID-19 Infection: An Assessment of a Physician Training Program | 2022 | USA | Observational pre- and post-interventional study | Emergency | SDM | Clinicians |  |
| [64] Niranjan S., Huand C., Dionne-Odom J. et al | Lay Patient Navigators’ Perspectives of  Barriers, Facilitators and Training Needs  in Initiating Advance Care Planning  Conversations With Older Patients  With Cancer | 2018 | USA | Interviews | Geriatrics | SDM | Lay navigators and oncology organisations |  |
| [65] Ledger U., Reid J., Begley A. et al | Moral distress in end-of-life decisions: A qualitative study of intensive  care physicians | 2021 | Northern Ireland | Qualitative interview study | Palliative | SDM | Clinicians |  |
| [66] Lindsay J., Dooley M., Martin J. (2014) | Reducing potentially inappropriate medications in palliative cancer patients: evidence to support deprescribing approaches | 2014 | Australia | Systematic Review | Palliative Medicine | DP | Clinicians |  |
| [67] Arends S., Francke AL., Jongerden IP. et al | Moral distress among nurses involved in life-prolonging treatments in patients with a short life expectancy: A qualitative interview study | 2022 | Netherlands | Interview study | Nursing | SDM | Nurses |  |
| [68] Guttmann K., Flibotte, J., Seitz, H. et al | Goals of Care Discussions and Moral Distress Among Neonatal Intensive Care Unit Staff | 2021 | USA | Prospective cohort study | Neonatal Intensive Care | SDM | Clinicians and wider healthcare staff |  |
| [69] Schulze-Westoff M., Groh A., Schroder S. et al | Potentially inappropriate medications according to PRISCUS list and FORTA (Fit fOR The Aged) classification in geriatric psychiatry: a cross‑sectional study. | 2022 | Germany | Cross-sectional study | Geriatrics | DP | Patients |  |
| [70] DiConti-Gibbs A., Chen K., Coffey C. et al | Polypharmacy in the Hospitalized Older Adult  Considerations for Safe and Effective Treatment | 2022 | USA | Summary review | Geriatrics | DP | Clinicians |  |
| [71] Hais J., Hellemans L., Laenen A. et al | The effect of a transitional Pharmacist Intervention in geriatric inpatients on hospital visits after discharge (ASPIRE): Protocol for a randomized controlled trial | 2021 | Belgium | Randomised controlled trial protocol | Geriatrics | DP | Pharmacists, patients |  |
| [72] Kalil R., Choi D., Geleris J. et al | Using clinical decision support tools to increase defibrillator deactivations in dying patients | 2022 | USA | Retrospective chart review | Palliative | Both | Clinicians and patients |  |
| [73] Gillespie R., Mullan J., Harrison L. | Exploring Older Adult Health Literacy in the Day-to-Day Management of Polypharmacy and Making Decisions About Deprescribing: A Mixed Methods Study | 2023 | Australia | Descriptive Statistics and Thematic Analysis | Geriatric Medicine | SDM | Clinicians and Patients |  |
| [74] Isautier J., Webster A., Lambert K. et al | Evaluation of the SUCCESS Health Literacy App for Australian Adults With Chronic Kidney Disease: Protocol for a Pragmatic Randomized Controlled Trial | 2022 | Australia | Protocol for RCT | Nephrology | SDM | Patients |  |
| [75] Sato A., Fujimori M., Shirai Y. et al | Assessing the need for a question prompt list that encourages end-of-life discussions between patients with advanced cancer and their physicians: A focus group interview study | 2022 | Tokyo | Interviews | Oncology | SDM | Clinicians, patients, family members |  |
| [76] Rennels C., Barnes D., Volow A. et al | PREPARE for your care and easy-to-read advance directives increase real-time goal concordant care | 2022 | USA | Surveys | Geriatrics | SDM | Patients |  |
| [77] Joseph-Williams, N., Newcombe, R., Politi, M. | Toward minimum standards for certifying patient decision aids: a modified delphi consensus process | 2014 | United Kingdom | Delphi Process | General Medicine | SDM | Patients |  |
| [78] Heen, A. H., Vandvik, P. O., Brandt, L. | Decision aids linked to evidence summaries and clinical practice guidelines: results from user-testing in clinical encounters | 2021 | Norway | Qualitative | General Medicine | SDM | Clinicians and patients |  |
