# Supplementary material for: Shared decision-making and deprescribing to support anti-thrombotic therapy (dis)continuance for persons living with cancer in their last phase of life: A realist synthesis
Source: PLoS Med. 2025 Aug 25;22(8):e1004663. doi: 10.1371/journal.pmed.1004663 (PMC12410886; doi:10.1371/journal.pmed.1004663)
Supplement: Attachment — Submitted filename: Response to reviewer and editorial comments.docx [file pmed.1004663.s009.docx]

| **Feedback** | **Response** |
| --- | --- |
| **Reviewer 1** |  |
| Thanks to the authors for addressing the reviewers' comments and clarifications/changes within the manuscript. The literature search/review section now is easier to follow and the discussion more concise. | Thank you. |
| **Reviewer 2:** |  |
| I feel that these have been satisfactorily answered, and where not, rationale has been given. | Thank you. |
| I did think that the bracketed point by Reviewer 4 "Discussing the kind of evidence that they have identified and which inform the review (and what kind of evidence that they may have expected that was NOT found in the evidence-base)" would have enriched the Discussion (without adding overly to Word Count), but this is a small point. | As acknowledged by the reviewer, this is a “small point” and, bearing in mind the importance of striking an appropriate balance in the Discussion that stimulates further debate but doesn’t detract from the focus of the review, our view is that the previous revisions to the strengths and limitations section address this point. |
| I see the authors have deferred to the Editorial team regarding the need to address two points specifically raised by Reviewers, and I likewise defer to Editorial team for same: | Please see below. |
| -Reviewer 1, point 19 on pg 4 of Response, regarding Table 4 references | For clarity, the original reviewer's comment was:  "For transparency it would be good to add the reference numbers to the papers listed in appendix three. It is frustrating trying to look up what has been numbered within the manuscript but not the table when wanting to find out which paper is discussed."  Our response to this was:  "Thank you for this suggestion, however the table of included studies in appendix 3 is organized according to the areas identified in Stage 1 (step A and B) and Stage 2, and not in order according to appearance in the manuscript. If the references were to be added to the table, they would not be listed in chronological order, and would still be inconvenient as the reader would need to scan the full table to find the specific paper linked to reference number. We could re-order the table in chronological order as the papers appear in the manuscript, however we would then loose their groupings according to the stages of the review. Currently the reader is required to go to the reference list to retrieve the paper based on the numerical reference in the body of the manuscript. This may be enough detail for most readers. We are happy to revisit this point with the journal editors should they recommend modifying the table."  We have now included the reference number for each paper in the included studies table for easier retrieval. |
| -Reviewer 2, point 10 on Response pg 6, regarding problems/potential solutions | For clarity, the original reviewer's comment was:  “For each of the thematic areas 1-3 (pages 11-19 in Reviewer copy), I think it would be useful to divide the test into 'problem's and 'potential solutions' as these are 2 aspects of the same theme.”  Our response to this was:  “Thank you for this valuable suggestion. We have reviewed the analysis on pages 11-19 to explore this option and feel that the presentation of data doesn’t lend itself well to dividing the analysis text in this way. The solution to the problem of medication continuance in the last phase of life is being developed through the on-going SERENITY study (https://serenity-research.eu/) in the form of a decision support tool to promote clinicians and patient conversations, and this is described in Table 4. However we would be happy to revisit this suggestion should the journal editors believe it to be important for presenting the findings.”  Whilst we appreciate this suggestion, the sections as they are currently organized do not lend themselves well to a dichotomous ‘problems’ and ‘solutions’ organization. Section one mainly describes the problems, and section three describes mainly solutions. We have, however added a number of CMO configurations throughout the sections to improve the readability and highlight the key mechanisms across the review findings. |
| **Reviewer #4** |  |
| The revised version has adequately responded to my review and concerns raised by other reviewers. | Thank you. |
| **Editor** | |
| GENERAL  * Please confirm that your title complies with to PLOS Medicine's style. Your title must be nondeclarative and not a question. It should begin with main concept if possible. "Effect of" should be used only if causality can be inferred, i.e., for an RCT. Please place the study design ("A randomized controlled trial," "A retrospective study," "A modelling study," etc.) in the subtitle (i.e., after a colon).  Suggestion: Shared decision-making and deprescribing in palliative care as support for anti-thrombotic therapy (dis)continuance for persons living with cancer in their last phase of life: A realist synthesis | Title revised to: Shared decision-making and deprescribing to support anti-thrombotic therapy (dis)continuance for persons living with cancer in their last phase of life: A realist synthesis |
| * Please revise for use of patient-centered language. Please note that patient-centered language is constructed with the use of post-modified nouns (e.g. 'patients with cancer’ (or similar) instead of ‘cancer patients’) putting the person first in the sentence structure. | We have now updated the manuscript to reflect more patient-centered language, replacing ‘cancer patients’ with ‘patients living with cancer’ |
| * Please ensure that all abbreviations are defined at first use throughout the text (including statistical abbreviations). Please also check figures and tables. | Checked and additions made where necessary. |
| * Please review your text for claims of novelty or primacy (e.g. 'for the first time', ‘novel’) and remove this language. | Done. |
| * Please ensure that tables and figures, including those in supplementary files, are appropriately referenced in the main text. | Done. |
| * We appreciate that "family" is considered an important factor in the synthesis. Have you given any thought or consideration to individuals who don't have family support, may have a legal guardian, etc.? | Our use of the term ‘family’ (instead of the unwieldy “significant others”) in the manuscript is inclusive of people who may be important to a patient living with cancer but who are not directly related. We recognise that the topic of our review is complex, and we have therefore taken the decision to refrain from adding complexity in the number and length of terms used unless strictly necessary. |
| * ll.748-751: We believe these are competing interest. Please make sure to include these in the relevant section in the online submission form. | We shall include these competing interests on the online submission form. |
| * If possible, we suggest presenting how many of the studies included details on either of one of the three main topics identified. | This has now been included – see additions to lines 380-384. |
| * Thank you for providing the RAMSES checklist. Please replace the page numbers with paragraph numbers per section (e.g. "Methods, paragraph 1"), since the page numbers of the final published paper may be different from the page numbers in the current manuscript. | Done. |
| * Regarding the points raised by Reviewer #2, we strongly encourage you to reconsider both of these points. We think that it should be clear which statements describe either a context or a mechanism and then an outcome, and that dividing the text into "problems" and "potential solutions" might be a useful structure for the text. However, we will not make these two points editorial requirements. | We have 11 embedded CMO statements in the analysis for improved clarity and reflecting clearer realist logic. Please see our response to Reviewer #2 above (p.2) regarding the reorganisation of the findings section according to problems and solutions. |
|  |  |
| ABSTRACT  * Please confirm that your abstract complies with our requirements, including providing all the information relevant to this study type https://journals.plos.org/plosmedicine/s/submission-guidelines#loc-abstract | Checked. Note: As the responses below required a number of additions that initially increased the abstract word count to more than 600 words, we have substantially revised the entire abstract (word count now 498 words). |
| * l.39, “Realist synthesis examining context-mechanism interactions.” – please change to a full sentence. | Done. |
| * l.43: Please list the databases. | Done. |
| * We think it would be useful if you could outline the types of articles covered by the 56 papers and 35 papers identified (i.e. how many were qualitative, how many were mixed methods, etc.). | Done. |
| * l.47: What does " genuine " mean in this context? Is this a scientific description? Please avoid emotive language. | Term removed. |
| * l.61: “actual, rather than feared” – Is this from a quote or is this your personal description? | “Actual, rather than feared” is a summary description of the synthesis appropriate for inclusion in an Abstract. |
| * How many citations were eventually included in the synthesis? | Done. |
| * Please outline the inclusion/exclusion criteria. | Done. |
| * Please ensure that all numbers presented in the abstract are present and identical to numbers presented in the main manuscript text. | Checked, done. |
| * In the last sentence of the Abstract Methods and Findings section, please describe the main limitation(s) of the study's methodology. | Done. |
| * l.68, we suggest changing to “that may causes devastating”. | Done. |
|  |  |
| AUTHOR SUMMARY  * Please refer to low or middle income countries rather than "developing countries" or "the Global South". Please refer to high income countries rather than "developed" or "Global North" countries. Please revise throughout the main text. | Done. |
| * If you agree, under "What did the researchers do and find?" we think you could add one or two bullet points explaining the theories in more detail. | Please see additions on lines 144-151. |
|  |  |
| INTRODUCTION  * Please ensure that the Introduction ends with a clear description of the study question or hypothesis. | This is included in Table 1. |
| * ll.132-134: We think that the review questions (Table 1) together with the definitions of study terms (Table 2) should be moved to the Methods section. | As the Methods section immediately follows the Introduction (at the end of which is Table 1, containing the review questions), our view is that moving the review questions to the Methods section is unnecessary and risks decreasing clarity (as the questions follow logically from the text in the Introduction).  We have moved Table 2 to the Methods section. |
|  |  |
| METHODS AND RESULTS  * When revising the manuscript, please keep in mind that PLOS Medicine has a broad audience that may not be familiar with the methodology of a realist synthesis. Please ensure that you provide a clear, structured, and easy-to-follow methodology that outlines the key steps in performing a realist synthesis.  Examples from PLOS One and PLOS Global Public Health:  https://doi.org/10.1371/journal.pgph.0003330  https://doi.org/10.1371/journal.pone.0285222 | We have revised the methodology section consistent with the suggested examples. |
| * In line with the comment above, we think that the structure of the Methods section could be improved, e.g. by subheadings (e.g. according to the sections outlined in the RAMESES checklist). | The methods section has now been re-organized and subheadings added according to Pawson’s five steps in a realist synthesis. |
| * l.152: Please remove claims of novelty or primacy. | Done. |
| * l.170, “widen the main search” – please explain in more detail and ensure to add a relevant reference to the full search terms. | We have reorganised the text in the search section so that this detail is clearly presented at a more logical point in the text (lines 254-257). |
| * l.175: Was there a set of certain exclusion criteria? Based on which criteria were the 180 citations excluded? Please clarify. | Exclusion criteria have now been described in lines 311-314. |
| * Figure 1: We suggest modifying Figure 1 to reflect (parts of) the PRIMSA flow diagram which also includes details on the databases searched, exclusion criteria etc. | We have now modified Figure 1 to reflect databases searched and exclusion criteria in addition to the other details. |
| * ll.195-204: These two sections seem rather disconnected from the main text. As you can see from a previous comment, we feel that the mention of inclusion and exclusion criteria should be part of the main text. Please revise. | The text has been re-organized with subheadings and exclusion criteria details have been added. |
| * l.210: “Members of the SERENITY research team and consortium recommended papers which were also included. – this statement is repetitive, please remove. | Done. |
| * l.210, “quota is filled” – what was the quota? | Stated and explained in lines 306-309. |
| * l.250: Please note that on line 250, there’s a ‘RESULTS’ heading while on 279 you added a ‘Findings’ heading. Please remove the latter. | Done. |
| * l.290, “catastrophic” – please consider whether the use of strong emotive language is necessary here. We suggest removing it. | Although we use the term ‘catastrophic’ in the clinical sense of ‘serious, untoward event’ and therefore disagree that this is “strong emotive language”, we can nevertheless see that some readers may interpret the term in the more general sense of “disaster”. We have therefore re-phrased to “severely distressing and disruptive” throughout the manuscript. |
| * ll.292-303: We suggest transforming the text into a table. | As the text directly links with Figure 2, we have incorporated it with this Figure. |
| * Figure 1: Please ensure to define all abbreviations used and to explain numbers 1-3. | We think this is referring to Figure 2, not Figure 1. By incorporating the text with Figure 2 (see row above), we have addressed this point. |
| * l.310: Please remove the numbering. You may use “The “Prescribing Inertia” Problem in End-of-Life Care” as a subheading. This also applies to the subsequent subheadings. | Done. |
| * l.389: Please note that you should consistently use the abbreviation "SDM" for shared decision making once you have introduced it. Please revise throughout. | Done. |
| * Table 4: Have you referenced Table 4 in the text? | Done. |
|  |  |
| DISCUSSION  * Please remove all subheadings. | We could not see any subheadings in this section to remove. |
| * We think the issues/topics you are discussing are important beyond anti-thrombotic Rx in terms of deprescribing at the end of life. We were wondering if you could be a little more explicit about the general interest of the question beyond anti-thrombotics for patients with cancer? | We agree and have added the following (lines 892-897): “The findings of our review can also inform the development of de-prescribing practices from a focus on identification (of medicines and of people with limited life expectancy) to an approach that encompasses de-prescribing SDM in palliative care. Such approaches are not limited to people living with non-malignant conditions, as they are relevant to the palliative care of people living with respiratory, cardiac, and neurological conditions in community, hospice, and hospital settings [Refs in manuscript]”. |
|  |  |
| General Editorial Requests  1) We ask every co-author listed on the manuscript to fill in a contributing author statement. If any of the co-authors have not filled in the statement, we will remind them to do so when the paper is revised. If all statements are not completed in a timely fashion this could hold up the re-review process. Should there be a problem getting one of your co-authors to fill in a statement we will be in contact. YOU MUST NOT ADD OR REMOVE AUTHORS UNLESS YOU HAVE ALERTED THE EDITOR HANDLING THE MANUSCRIPT TO THE CHANGE AND THEY SPECIFICALLY HAVE AGREED TO IT. | We acknowledge this. No changes have been made to authorship. |
| 2) Please ensure that the paper adheres to the PLOS Data Availability Policy (see http://journals.plos.org/plosmedicine/s/data-availability), which requires that all data underlying the study's findings be provided in a repository or as Supporting Information. For data residing with a third party, authors are required to provide instructions with contact information for obtaining the data. PLOS journals do not allow statements supported by "data not shown" or "unpublished results." For such statements, authors must provide supporting data or cite public sources that include it. | We acknowledge this. |
| 3) Please review your reference list to ensure that it is complete and correct. If you have cited papers that have been retracted, please include the rationale for doing so in the manuscript text, or remove these references and replace them with relevant current references. Any changes to the reference list should be mentioned in the rebuttal letter that accompanies your revised manuscript. | Done. |
