# Supplementary material for: Shared decision-making and deprescribing to support anti-thrombotic therapy (dis)continuance for persons living with cancer in their last phase of life: A realist synthesis
Source: PLoS Med. 2025 Aug 25;22(8):e1004663. doi: 10.1371/journal.pmed.1004663 (PMC12410886; doi:10.1371/journal.pmed.1004663)
Supplement: Attachment — Submitted filename: Response to reviewer comments R2.docx [file pmed.1004663.s008.docx]

| **Feedback** | **Response** |
| --- | --- |
| **Reviewer 1** |  |
| After reading the manuscript I feel its title needs adjustment, given the research questions and the discussed literature mainly cover deprescribing and SDM at the end of life in general. The method section states, 'the analysis focussed on locating causal insights in the published literature regarding deprescribing for patients in the last phase of life and shared decision-making in that context' (without specifying what that context may be). There is some focus on ATT but the program theory closely resembles those developed by other realist syntheses around deprescribing and SDM without explaining ATT specific contexts and mechanisms (some nuances are mentioned in the discussion). While it will be applicable to and support the development of SDM support tools for discontinuation of ATT it seems not reflective of the chosen title. | We agree and have revised the title to: How can current evidence on shared decision-making and deprescribing in palliative care support anti-thrombotic therapy (dis)continuance for persons living with cancer in their last phase of life? A realist synthesis  Throughout our response, ‘L’ refers to the line number of the R2 manuscript (tracked changes version). |
| 1. L 116 - impede not impeded? | Corrected to ‘impede’. |
| 2. L 116 - refining theories - as theory development has not yet been mentioned, 'refining' seems out of context here. | Changed to ‘By developing, testing and refining…’ (L147) |
| 3. L 123 - 'illuminating (that word always sounds a bit like AI) the importance of contextual factors' doesn't quite align with the end of the sentence 'and reasons why it can fail' which in a realist sense would usually describe mechanisms. | Changed to ‘In understanding how context impacts the mechanisms associated with optimal deprescribing, the analysis provides new programme theories as groundwork for new service innovations.’ (L154-156) |
| 4. L 145 - 'The main search for literature was developed and conducted' - this sound as if the information specialist started afresh, did they use/ build on the initial scoping searches, adapting them to each database? I am curious why so many of them were searched, wouldn't it be highly likely that the approach to selection, RCQ and expert solicitation, would have yielded the most relevant papers by searching the databases most relevant to the field of investigation? | Thank you for highlighting that additional clarity might be needed here. We have revised the wording in the "Literature search and article screening" section (L160-203) to clarify that the information specialist conducted both scoping and main searches and that results from scoping searches and other sources informed the development and testing of the main search.  The "main search" in this realist synthesis is comparable to the comprehensive search for empirical evidence in a traditional systematic review (see Booth 2020 for more details of the different components that might be used in a realist search) and so used a variety of databases to build a broad set of literature that was used in RCQ screening for Step A and also enabled targeted searching within this set of literature for Step B. |
| 5. L 177 - throughout it is not clear whether evidence for shared decision making AND deprescribing was evaluated, OR either or. The search strategy was formulated with OR, but shared decision making to achieve deprescribing is often a particularly difficult process to establish. At this point in the manuscript, I was under the assumption the synthesis tries to establish Cs and Ms to achieve deprescribing of ATT as a shared decision. | The reviewer is correct in assuming that the synthesis aimed to establish Cs and Ms to achieve deprescribing of ATT as a shared decision. However, there was no literature addressing this exact question, which is why we broadened the search in the areas of shared decision-making in palliative care and deprescribing. The ‘OR’ in the search string was intentional because we wanted to capture rich insights in papers either in shared decision making (SDM) or deprescribing and then synthesize these finding for application to the review questions specifically. |
| 6. Given the complexity of the topic and overall search it may be difficult to outlay it with exacting clarity (without asking readers to go to appendix 3) but it wasn't clear why some terms were included without reference to deprescribing, e.g. moral distress - although the chosen papers relate to end-of-life care, in the manuscript it isn't clear that this was the case. It may be worthwhile providing a clearer picture and rationale of how the searches (and later choices of included papers) relate to each other, in addition to the listing of the stepwise approach taken. | The search terms from phase one represented the broader search of the literature that was used to extrapolate insights to the specific question of ATT deprescribing for patients living with cancer in the last phase of life. This was done because of a lack of availability of research papers pertaining to the exact scope of the review which was to understand how shared decision-making works in the context of ATT deprescribing for end-of-life cancer care  The search terms in the second phase were refined after input from stakeholders at stakeholder meetings. This included both practitioner and patient/public involvement groups. This is described in figure 1. A line has been added to the stakeholder consultation section to further describe this (L278-279). |
| 7. It may also be beneficial at this point and in the findings to state clearly that due to the sparsity of literature dealing specifically with deprescribing of ATT in the patient group of interest focus had to be adjusted and extrapolation took place. That would probably avoid confusion among some readers (mentioned below). | Text added to clarify this. |
| 8. Overall, it may be easier for readers to follow the program theory statements if they were placed after the narrative summary of the literature. This would answer a number of questions I had when reading through them following the current order. | Thank you for this suggestion. We have moved the programme theory statements to the end of the narrative summary of the literature |
| 9. L 227 - it would be good to outline how the areas of prescribing inertia and training for decision support were established. For example, the first is a well-established concept in the general deprescribing literature, was it only drawn from the papers included in the synthesis? | Yes, these were drawn from the literature and used to formulate a framework for the analysis. Prescribing inertia was a useful concept to help unpack the context. This concept emerged through our data immersion. See description (L221-230). |
| 10. L 237 - It would be helpful for the if - then statements to be clearly signposted as they are not configured as CMOCs (as described in the methods), the 'then' at times stands in for the 'because' of a mechanism. Which ones relate to contexts, which ones to mechanisms? CMOCs may not be necessary as the outcome for all would be deprescribing of ATT?  11. Some statements seem to describe either a context or a mechanism and then an outcome, which again was confusing in terms of realist logic. For example, 1c seems to describe a problem of mechanisms, clinicians feeling uncertain etc. leading to an outcome of persistent inertia. As a context it probably would be better formulated as the fact that there is uncertainty about risk-benefit. In the absence of evidence-based guidance clinicians negotiate unmeasurable harms and benefits (rather than 'feeling' uncertain there is real uncertainty) which leads to the maintenance of the status quo, or inertia. | Thank you for your attention on the matter of the programme theories (comments #10 and #11 are closely-linked). We appreciate the reviewer’s rationale for configuring CMOCs consistently, but believe that is important to prioritized the readability of the if…then statements over consistent use of the ‘if..then’ format (‘if (context)…then (mechanism)’). This is to ensure the readability of the statements, each of which still provides an explanatory view on the data which is the criterion needed for the realist approach (in line with the RAMESES reporting standards).  Regarding programme theory 1c, we would argue that ‘clinicians feeling uncertain’ is context, even if it is a ‘feeling’. This is a common confusion with using the realist approach. What characterizes context is that it exists apart from, or outside of the service innovation. Clinical uncertainty is high in the context of end-of-life care generally (context). This feeling of uncertainty would be classified as a mechanism if it is in response to a service innovation targeting the issue at hand. So, for example if clinicians feel increased uncertainty while using a PIMs list algorithm, then it would be a mechanism that explains success or failure of the application.  We also decided to include the CMO thinking in figure 2 to gain ensure we are not losing the reader given the level of complexity involved in the analysis.  We are happy to revisit this question should the editors feel it to be important to depict the If..then statements in a different way. |
|  | Thank you for your attention on the matter of the programme theories and appreciate the reviewer’s rationale for identifying the CMO thinking within the programme theories.. |
| 12. For the domain of shared decision making, it would also be good to discern that 2a and b relate to mechanisms whereas statements 3 a and b describe training / education and the access to decision support as enabling contexts for those Ms. | Thank you for this suggestion. Indeed 1a-1c relate to contexts whereas the remainder 2a-3b relate to mechanisms and this is depicted in Figure 2 |
| Narrative summary:  13. L 391 & 405 I am not sure that the mention of one paper supporting a concept warrants the use of the term 'literature'. | This has been modified to ‘One paper reflected the…’ |
| 14. L 417 The statement of 'Shared decision-making across interprofessional staff teams has been shown to counter prescribing inertia' would need a reference as the literature (the way it is described) within the following paragraph does not support or evidence it. | Please see edits lines 492-501 for improved clarity |
| 15. L 454 While the Beers or STOPP/START criteria may assist in making decisions on deprescribing for older people in general, they are not tailored to end-of-life decisions in cancer patients (many of whom are not old). It is not clear whether they are discussed as general examples of the presence of decision support tools achieving deprescribing at a higher rate than their absence or as useful in making decisions to deprescribe ATT in cancer patients. | The inclusion of Beers and Stopp/Start was to provide causal insights about the use of PIMs lists that could be extrapolated to the area of end-of-life care and ATT deprescribing. It is correct to say that these lists are not specific to the scope of the review, however the SERENITY project is building decision support specific to ATT deprescribing which is supported by a general view on decision support tools in palliative care. |
| 16. L 544 This flows on into table 3. I thought this realist review was supposed to underpin SDM support tools for the deprescribing of ATT in cancer patients, whereas the recommendations in the table seem to refer to something more generic. While many of them will be exactly the same for something more specific, what would adding a PIMS list contribute when targeting a very specific group of medicines? | Thank you for this observation. You are correct in stating that the statements in Table 4 could be applied generally across decision support applications (not specific to ATT at end of life). However, these statements are meant to be read by clinicians and researchers working specifically in the area of ATT deprescribing and can benefit from these broad considerations as a starting point.  Having said this, we have modified the title of the table to include ‘for ATT deprescribing’ |
| Discussion:  17. I found the discussion difficult to follow, partly because some sentences are very long, e.g. L 557-561, 558-592 (breaking them up would assist readers), and because it seems to repeat some of the findings but also discuss new ones. It reads a bit like everyone may have been adding points they thought needed mentioning, which interrupts cohesion and makes it quite long for a realist review, in which the details of the program theory would be the main discussion points.  18. It also reflects what I perceive as the shifting between general deprescribing at the end of life and that of ATT throughout the manuscript. It raises issues and arguments which I would have expected in the findings. For example, I wonder whether the discussion points at L 578 (if supported by literature) shouldn't be included in the PT, as ATT as primary or secondary prophylaxis or treatment of thromboembolic events and whether anticoagulants or antiplatelet agents are in use would greatly influence clinicians' and people's reasoning around its (de)prescribing. As such the statements starting in L 623, that the review addresses the complexity of the architecture of deprescribing of a specific class of medications, seems a stretch too far, particularly as conclusions have been reached mainly by extrapolation rather than evidence. | The discussion section has been revised and shortened to improve its readability. Please see pages L634-757 for revisions. |
| 19. For transparency it would be good to add the reference numbers to the papers listed in appendix three. It is frustrating trying to look up what has been numbered within the manuscript but not the table when wanting to find out which paper is discussed. | Thank you for this suggestion, however the table of included studies in appendix 3 is organized according to the areas identified in Stage 1 (step A and B) and Stage 2, and not in order according to appearance in the manuscript. If the references were to be added to the table, they would not be listed in chronological order, and would still be inconvenient as the reader would need to scan the full table to find the specific paper linked to reference number. We could re-order the table in chronological order as the papers appear in the manuscript, however we would then loose their groupings according to the stages of the review. Currently the reader is required to go to the reference list to retrieve the paper based on the numerical reference in the body of the manuscript. This may be enough detail for most readers. We are happy to revisit this point with the journal editors should they recommend modifying the table. |
| **Reviewer 2:** |  |
| 1.      Abstract: I thought the potential mechanisms outlined underpinning SDM were helpful and could be considered/trialled by clinicians and managers. In fact, I think these should also be explicitly included in the main text- otherwise, if I search e.g. 'empowerment' or 'investment' in the main text, these don't show up at all, and I have to wade through the Discussion to see where the authors mentioned these issues. | Please see edits L287-296. |
| Intro:  2.      Good overview of issue. | Thank you. |
| 3.      Paragraph 2 first few points would benefit from some citations to back up statements (even though intuitively I agree). There is some literature around deprescribing towards end of life and in other terminal conditions (e.g. dementia) which may be of relevance here. | We agree – citations added (5-7, L112-115). |
| 4.      The boxes were helpful for clarity. | Thank you |
| Methods:  5.      "RCQ is a screening strategy in which papers are screened starting from the most recent date and working in reverse chronology until a quota is filled." Please include planned a priori quota. | See revisions (L213-216) |
| 6.      Please include rough numbers for consumer groups included in "The other two meetings were held through a patient and public involvement group, comprising  people living with, and supporting people with cancer, as well as members of the public at large." | Appendix 3 details these numbers. Providing ‘rough numbers’ for the patient groups in the main text would require also providing numbers for the professional meetings and this not only adds more text to the already dense manuscript but we feel this level of detail is better suited for the appendix as it is unclear what meaning would be ascribed to the number of people attending the meetings. |
| Results: 7.      "Four key content areas organised the dataset." Can the authors mention how these were determined- from which part of the described methodological process? | Please see revisions (L275-282). |
| 8.      Figures are helpful for clarity. Small pint: please give legend for abbreviations with the figure (e.g. PIMS, eMR for Figure 2- although I know what these are, some readers may not and they are defined later in the text from current siting of Figure 2. | The terms ‘PIMs list’ and ‘EMR’ have been added to the definition of terms table which appears before the figure. |
| 9.      "The findings * are organised across three areas (prescribing inertia, shared decision-making, 228 education, training and decision support for shared decision-making) and seven sub-areas 229 (Figure 2)." *Pleas add 'of the literature review' if I have interpreted correct. | Please see revision on line 285. |
| 10.     For each of the thematic areas 1-3 (pages 11-19 in Reviewer copy), I think it would be useful to divide the test into 'problem's and 'potential solutions' as these are 2 aspects of the same theme. | Thank you for this valuable suggestion. We have reviewed the analysis on pages 11-19 to explore this option and feel that the presentation of data doesn’t lend itself well to dividing the analysis text in this way. The solution to the problem of medication continuance in the last phase of life is being developed through the on-going SERENITY study (<https://serenity-research.eu/>) in the form of a decision support tool to promote clinicians and patient conversations, and this is described in Table 4. However we would be happy to revisit this suggestion should the journal editors believe it to be important for presenting the findings. |
| 11.     Re SDM tool: "A SDM tool should educate on and explain to clinicians the problem with prescribing inertia and the importance of engaging in discussions about deprescribing with patients and families" Shouldn't it also educate patients/carers? It should be used by the clinician and patient/carer together, so both will need education on its use/what it can achieve. | Thank you, this has been corrected |
| Discussion: 12.     "The analysis covers three theory areas": if these were developed from the analysis, and not pre-determined, this should be highlighted. | Please see revision on L635-636. |
| 13.     "The findings of this review relate to other realist studies on deprescribing such as the TAILOR 606 evidence synthesis [57] the deprescribing rainbow model [77] and MEMORABLE realist review 607 [78],"- It would be good if the patient cohorts included in each of these could be bracketed, for reader's easy reference. | We have clarified that each of these studies focused on deprescribing for older people. |
| 14.     Please see above re summary findings in Abstract, not mirrored explicitly in main text. | See edits L287-296. |
| **Reviewer #3:** |  |
| Thanks so much for this excellent review of deprescribing. Though it is targeted about antithrombotic drugs it delves deeply into related contextual issues of deprescribing in general, shared decision-making and EMR-generated triggers to encourage advance care planning and goals of care discussions ("Serious Illness Conversations"). My only minor criticism is that the title focusing on ATT does not do it full justice. With good key words I hope however that it will be easily findable when searching general terms. | Thank you for your positive comments. We have also amended the title to better reflect the review scope and purpose. |
| **Reviewer #4** |  |
| Thank you for the opportunity to review this manuscript. This is a realist synthesis on how when and what kind of settings should clinicians discuss about continuing or discontinuing anti thrombotic medication. This is a relatively unique setting for the application of the realist synthesis because it has not been well applied yet in clinical decision-making settings such as this. However, the methodology lends itself well to the setting being studied. Shared decision-making between clinicians and caregivers or patients is a very important area for application of realist methodology because of various considerations at the individual, patient and caregiver characteristics level, at the history and nature of relationship between the caregiver and provider, as well as various workplace and other environmental considerations within the clinical settings where such conversations occur. Given the multilevel nature of these interactions shared decision making cannot be studied merely from the angle of effectiveness of the educational content provided by the clinician, but rather needs to take into consideration multilevel interactions across various domains which indeed span the discipline of psychology, communication, and organisational sciences. Such interdisciplinary approaches could help clinicians better understand what kind of shared decision making approaches work for which patients and in what settings and for the latter, the realist approach.   My comments here in should be interpreted from a place of expertise in the methodology. I am not an expert in the clinical area being studied here, although I am a trained clinician myself and continue a small portion of my time on clinical care.  I compliment the author team for carefully bringing the strengths of the realist approach to this area of clinical enquiry in shared decision-making. Indeed, I believe the overall approach they have taken for studying shared decision making in the context of discontinuation of ATT in end of life Cancer care can be expanded to be applied for shared decision-making conversations in various other clinical settings as well. I find the application of the realist approach to have been done using state of the Art guidance relevant for this. The development of if - then propositions which explain the phenomenon being studied and form a hypothetical basis for refining using observation and quality of data is well presented.  The authors do not substantively discuss the geographical and social settings that are represented in the 35 papers that they have retained. This has two important implications. One is related to relative lack of literature from Limited resource across the World, predominantly in LMIC settings, but also in Limited resource settings in high income countries as well. The second concern is the fact that a lot of social science, theorising is done in high income and well-resourced settings which includes a probable bias in the program theories and mechanisms that arise out of such work. Indeed, this bias has been well pointed out in literature as well. Discussing the kind of evidence that they have identified and which inform the review (and what kind of evidence that they may have expected that was NOT found in the evidence-base), and critically sharing in the discussion section/limitations section what implication this has on their findings is something useful to consider and is offered here as an optional suggestion to the authors. If this is not feasible, it may be important to qualify their assertion in the conclusion that their synthesis is based on an "…international literature base" (the question being how international is the evidence-base?). | Thank you for your comments recognising the distinctiveness and strengths of our realist synthesis. Your observations regarding our claim of an “international literature” (and the implications of uncritical development of programme theory with a Global North bias) are pertinent given that just one included source related to a country in the Global South. In light of other reviewers’ comments about honing the Discussion (rather than adding elements), we don’t think that this manuscript is the place to go into detailed reflection about this. However, as suggested, we have revised ‘international’ to ‘Global North’ (or ‘Europe’, where applicable) and acknowledged in the strengths & limitations that “we did not pursue additional routes of programme theory development to include knowledge from the Global South” (L758-766). |
| **Editor** | |
| We ask every co-author listed on the manuscript to fill in a contributing author statement, making sure to declare all competing interests. If any of the co-authors have not filled in the statement, we will remind them to do so when the paper is revised. If all statements are not completed in a timely fashion this could hold up the re-review process. If new competing interests are declared later in the revision process, this may also hold up the submission. Should there be a problem getting one of your co-authors to fill in a statement we will be in contact. Please do not add or remove authors without first discussing this with the handling editor. You can see our competing interests policy here: http://journals.plos.org/plosmedicine/s/competing-interests. | Noted. |
| Please upload any figures associated with your paper as individual TIF or EPS files with 300dpi resolution at resubmission; please read our figure guidelines for more information on our requirements: http://journals.plos.org/plosmedicine/s/figures. While revising your submission, please upload your figure files to the PACE digital diagnostic tool, https://pacev2.apexcovantage.com/. PACE helps ensure that figures meet PLOS requirements. To use PACE, you must first register as a user. Then, login and navigate to the UPLOAD tab, where you will find detailed instructions on how to use the tool. If you encounter any issues or have any questions when using PACE, please email us at PLOSMedicine@plos.org. | Checked using PACE and uploaded to Editorial Manager. |
| Please ensure that the paper adheres to the PLOS Data Availability Policy (see http://journals.plos.org/plosmedicine/s/data-availability), which requires that all data underlying the study's findings be provided in a repository or as Supporting Information. For data residing with a third party, authors are required to provide instructions with contact information (web or email address) for obtaining the data. Please note that a study author cannot be the contact person for the data. PLOS journals do not allow statements supported by "data not shown" or "unpublished results." For such statements, authors must provide supporting data or cite public sources that include it. | Compliance with PLOS Data Availability Policy checked. |
| We expect all researchers with submissions to PLOS in which author-generated code underpins the findings in the manuscript to make all author-generated code available without restrictions upon publication of the work. In cases where code is central to the manuscript, we may require the code to be made available as a condition of publication. Authors are responsible for ensuring that the code is reusable and well documented. Please make any custom code available, either as part of your data deposition or as a supplementary file. Please add a sentence to your data availability statement regarding any code used in the study, e.g. "The code used in the analysis is available from Github [URL] and archived in Zenodo [DOI link]" Please review our guidelines at https://journals.plos.org/plosmedicine/s/materials-software-and-code-sharing and ensure that your code is shared in a way that follows best practice and facilitates reproducibility and reuse. Because Github depositions can  be readily changed or deleted, we encourage you to make a permanent DOI'd copy (e.g. in Zenodo) and provide the URL. | Not applicable |
| Please ensure that the study is reported according to the RAMESES guideline (https://www.equator-network.org/reporting-guidelines/rameses-publication-standards-realist-syntheses/) and include the completed RAMESES checklist as Supporting Information. When completing the checklist, please use section and paragraph numbers, rather than page numbers. Please add the following statement, or similar, to the Methods: "This study is reported as per RAMESES guideline (S1 Checklist)." | RAMESES checklist included (Appendix 5) with appropriate statement in Methods section. |
|  |  |
| FORMATTING - GENERAL |  |
| * Abstract: Please structure your abstract using the PLOS Medicine headings (Background, Methods and Findings, Conclusions). Please combine the Methods and Findings sections into one section. | Done. |
| * At this stage, we ask that you include a short, non-technical Author Summary of your research to make findings accessible to a wide audience that includes both scientists and non-scientists. The Author Summary should immediately follow the Abstract in your revised manuscript. This text is subject to editorial change and should be distinct from the scientific abstract. Ideally each sub-heading should contain 2-3 single sentence, concise bullet points containing the most salient points from your study. In the final bullet point of 'What Do These Findings Mean?', please include the main limitations of the study in non-technical language. Please see our author guidelines for more information: https://journals.plos.org/plosmedicine/s/revising-your-manuscript#loc-author-summary. | Done. |
| * Please express the main results with 95% CIs as well as p values. When reporting p values please report as p<0.001 and where higher as the exact p value p=0.002, for example. Throughout, suggest reporting statistical information as follows to improve clarity for the reader "22% (95% CI [13%,28%]; p</=)". Please be sure to define all numerical values at first use. | Not applicable |
| * Please include page numbers and line numbers in the manuscript file. Use continuous line numbers (do not restart the numbering on each page). | Done. |
| * Please cite the reference numbers in square brackets. Citations should precede punctuation. | Done. |
|  |  |
| FIGURES AND TABLES  * Please provide titles and legends for all figures and tables (including those in Supporting Information files). | Done. |
| * Please define all abbreviations used in each figure/table (including those in Supporting Information files). | Done. |
| * Please consider avoiding the use of red and green in order to make your figure more accessible to those with color blindness. | Done. |
|  |  |
| SUPPLEMENTARY MATERIAL  * Please note that supplementary material will be posted as supplied by the authors. Therefore, please amend it according to the relevant comments outlined here.  * Please cite your Supporting Information as outlined here: https://journals.plos.org/plosmedicine/s/supporting-information | Done. |
|  |  |
| REFERENCES  * PLOS uses the numbered citation (citation-sequence) method and first six authors, et al.  * Please ensure that journal name abbreviations match those found in the National Center for Biotechnology Information (NCBI) databases (http://www.ncbi.nlm.nih.gov/nlmcatalog/journals), and are appropriately formatted and capitalised.  * Where website addresses are cited, please include the complete URL and specify the date of access (e.g. [accessed: 12/06/2023]).  * Please also see https://journals.plos.org/plosmedicine/s/submission-guidelines#loc-references for further details on reference formatting. | Done. |
